# Interventional Clinical Trials in Metastatic Pulmonary Large-Cell Neuroendocrine Carcinoma: A Systematic Review of Prospective, Interventional Trials

**DOI:** 10.3390/cancers18060964

**Published:** 2026-03-17

**Authors:** Elettra Merola, Maria Pina Dore, Giuseppe Fanciulli

**Affiliations:** 1 Department of Medicine, Surgery and Pharmacy, University of Sassari, 07100 Sassari, Italy; mpdore@uniss.it (M.P.D.); gfanciu@uniss.it (G.F.); 2 Department of Medicine, Baylor College of Medicine, One Baylor Plaza Blvd, Houston, TX 77030, USA; 3 Unit of Endocrinology, AOU Sassari, Viale San Pietro 43b, 07100 Sassari, Italy

**Keywords:** lung, large cell neuroendocrine carcinoma, systematic review, clinical trials

## Abstract

**Background/Objectives:** Pulmonary neuroendocrine neoplasms comprise a heterogeneous group of epithelial lung tumors characterized by varying degrees of differentiation and biological aggressiveness. Among high-grade types, large cell neuroendocrine carcinoma (LCNEC) is an uncommon but clinically significant entity, representing approximately 3% of lung cancers, with the optimal first-line therapeutic approach remaining uncertain. Our aim was to evaluate the available prospective therapeutic evidence in patients with advanced or metastatic pulmonary LCNEC, defining the current evidence and identifying key gaps to inform future research. **Methods**: A systematic literature search was conducted using PubMed, Scopus, and Web of Science (last update: 9 August 2025). Endpoints included the evaluation of clinical outcomes from interventional trials and safety. **Results**: Overall, 2139 records were identified through the database search and handsearching. After removal of duplicates and non-eligible records, only 4 prospective, non-randomized studies were eligible for the systematic review. Due to the risk of bias and substantial methodological variability, a meta-analysis could not be reliably performed. Three trials investigated first-line platinum-based chemotherapy regimens, while only one study evaluated immunotherapy in lung LCNEC. Overall clinical outcomes were modest, with benefits appearing limited in duration, with median PFS consistently <6 months and median OS rarely exceeding one year. **Conclusions**: The findings of this review underscore a persistent and substantial evidence gap in the management of advanced pulmonary LCNEC. Closing this gap will require coordinated international collaborations and innovative trial designs, including molecular analysis as a driver for patient management. Without such efforts, the prospect of personalized medicine for lung LCNEC will remain far from reality.

## Simple Summary

Large cell neuroendocrine carcinoma (LCNEC) of the lung is a rare and aggressive form of lung cancer, accounting for approximately 3% of cases. Despite its clinical relevance, the optimal treatment strategy for advanced or metastatic LCNEC remains unclear. We conducted a systematic review of prospective interventional clinical trials to evaluate the available therapeutic evidence. Only four non-randomized studies met inclusion criteria. Most trials investigated platinum-based chemotherapy in the first-line setting, while immunotherapy was evaluated in only one study. Clinical outcomes were modest, with progression-free survival typically shorter than six months and overall survival rarely exceeding one year. The limited number of trials, small sample sizes, and methodological variability highlight a significant evidence gap. These findings emphasize the urgent need for international collaboration, innovative clinical trial designs, and integration of molecular profiling to improve treatment strategies and enable personalized care for patients with lung LCNEC.

## 1. Introduction

Pulmonary neuroendocrine neoplasms (NENs) comprise a heterogeneous group of epithelial lung tumors characterized by varying degrees of differentiation and biological aggressiveness. This spectrum includes well-differentiated neoplasms, namely typical and atypical carcinoids, as well as poorly differentiated high-grade neuroendocrine carcinomas (NECs). Among NECs, small cell lung carcinoma (SCLC) accounts for 15% of newly diagnosed lung cancers compared with 3% for large cell neuroendocrine carcinoma (LCNEC). Throughout this review, the definition LCNEC is used according to the diagnostic criteria of the 2021 WHO Classification of Thoracic Tumors, which defines LCNEC as a high-grade pulmonary neuroendocrine carcinoma with neuroendocrine morphology, high mitotic activity, and immunohistochemical evidence of neuroendocrine differentiation (Supplementary Table S1) [1,2].

While typical and atypical carcinoids account for a small proportion of lung malignancies and are generally associated with favorable clinical outcomes, lung NECs represent the most aggressive end of this spectrum and are characterized by rapid growth, early metastatic dissemination, and poor prognosis [3,4]. Indeed, SCLC and LCNEC exhibit markedly lower survival rates compared with carcinoid tumors, even when diagnosed at limited stages, and are associated with high rates of nodal and distant metastases [5].

Surgical resection, with or without adjuvant chemotherapy, is reserved for a selected subset of lung NECs with localized, technically resectable disease [6]. In the setting of extensive-stage disease, representing more than half of the newly diagnosed cases, systemic therapy remains the mainstay of treatment, but durable long-term survival remains uncommon [7]. Although therapeutic strategies for pulmonary NECs have evolved in recent years, current standard treatments remain suboptimal, and management in a multidisciplinary setting is recommended [8].

The optimal first-line therapeutic approach for metastatic lung NECs remains uncertain, especially for LCNEC, due to their rarity [5,9,10]. Consequently, therapeutic strategies for pulmonary LCNEC have historically been extrapolated from SCLC, with platinum-based chemotherapy representing the most adopted systemic treatment [4,11]. Although LCNEC and SCLC are both high-grade NECs and may share aggressive clinical behaviour, LCNEC is typically characterized by larger polygonal cells, more abundant cytoplasm, visible nucleoli, and organoid neuroendocrine architecture, in contrast to the smaller cells, scant cytoplasm, finely granular chromatin, and diffuse sheet-like growth more typical of SCLC. At the molecular level, SCLC is classically defined by near-universal *TP53* and *RB1* co-inactivation, whereas LCNEC is more heterogeneous and includes both SCLC-like subsets with *TP53/RB1* co-alteration and NSCLC-like subsets enriched for *STK11*, *KEAP1*, *KRAS*, and related alterations [12,13,14]. Clinical outcomes remain unsatisfactory, with limited durability of response and poor survival reported across available series [3]. The absence of disease-specific prospective trials has contributed to persistent uncertainty regarding optimal treatment selection, while diagnostic complexity and frequent misclassification with other high-grade NENs have further hindered the generation of robust clinical evidence [5].

The limited representation of high-grade NENs in interventional clinical trials has been increasingly recognized as a major barrier to the development of evidence-based therapeutic strategies for these aggressive malignancies [15,16]. This gap is particularly relevant for pulmonary NECs, where clinical management often relies on treatment paradigms extrapolated from other tumor types. Although recent years have witnessed a growing interest in exploring novel systemic approaches for lung NECs, including the evaluation of immunotherapy and targeted agents, the extent and methodological robustness of prospective evidence specifically addressing LCNEC remain uncertain [5,17]. As a result, the available data supporting therapeutic decision-making in this setting are still limited, highlighting the need for a clearer characterization of the current prospective evidence base.

Given the rarity of LCNEC and the absence of randomized controlled trials specifically dedicated to this disease, prospective interventional studies currently represent the most robust level of therapeutic evidence available. We therefore conducted a systematic review of interventional clinical trials to evaluate the available prospective therapeutic evidence in patients with advanced or metastatic pulmonary LCNEC, focusing on efficacy, survival outcomes, and safety, with the aim of defining the current evidence base and identifying key gaps to inform future research.

## 2. Materials and Methods

The present systematic review was aimed at evaluating the clinical outcomes (including objective response rate, ORR; disease control rate, DCR; survival data) as the primary endpoints, and safety as the secondary endpoint, observed in patients with sporadic, advanced/metastatic LNECs of the lung treated with the various therapeutic approaches reported in clinical trials.

The review protocol was registered in the PROSPERO database (CRD420251128910). A comprehensive systematic search was conducted across the PubMed, Scopus, and Web of Science databases, with the most recent update on 9 August 2025. Detailed search strategies specific to each database are reported in Supplementary Material S1. In order to include only prospective interventional studies, filters for randomized controlled trials and clinical trials were applied. Findings from this literature search related to gastro-entero-pancreatic NENs have already been published in a separate report [16].

Exclusion criteria related to tumor type were: non-neuroendocrine neoplasms; grade 1 or grade 2 neuroendocrine tumors; hereditary cancer syndromes; primary sites other than pulmonary; and SCLC.

Regarding study design, we excluded: reviews and meta-analyses; retrospective studies; preclinical or laboratory investigations; case reports and small case series; correspondence and guidelines; clinical trials with fewer than 10 lung NENs, in order to minimize the risk of small-sample bias and the over-representation of uncontrolled case-series evidence; conference abstracts or unpublished data; and non-interventional prospective studies.

Study selection began with screening of titles and abstracts, followed by full-text assessment of articles deemed potentially eligible. The process was independently led by two authors (EM and GF) using Rayyan (Rayyan Systems Inc., Cambridge, MA, USA) [18], with a third author (MPD) serving as a tiebreaker in case of disagreement. The indications of Preferred Reporting Items for Systematic Reviews and Meta-Analyses (PRISMA) 2020 guidelines [19] were followed. After the identification of eligible studies, data extraction for quantitative analysis was independently performed by EM and GF.

All studies excluded during the selection process were systematically documented together with the corresponding reasons for exclusion, in order to ensure transparency and reproducibility of the screening procedure. Only data available in the published reports were considered for the analysis, and no attempts were made to contact study investigators to obtain additional or unpublished information.

From each eligible study, the following variables were extracted: the name of the first author, publication type and year, total sample size, and the number of patients diagnosed with LCNEC. In addition, detailed information regarding the treatment strategies investigated in each study was collected, including the characteristics of the therapeutic regimen administered and the main reported clinical outcomes. These data included efficacy, survival, and safety results as described in the original publications.

Efficacy endpoints included ORR and DCR, while survival outcomes comprised progression-free survival (PFS) and overall survival (OS). Safety was assessed based on the incidence and severity of adverse events reported in the individual trials, as documented in the study results.

Risk of bias was independently evaluated by two reviewers (EM and GF) using the ROBINS-I tool for non-randomized studies [20,21]. Any discrepancies in assessment were resolved by consensus, with a third reviewer (MPD) involved when necessary.

Language editing of the manuscript and preparation of the graphical abstract were performed with the assistance of an artificial intelligence-based language model (ChatGPT 5, OpenAI).

## 3. Results

### 3.1. Study Selection

**Figure 1:**
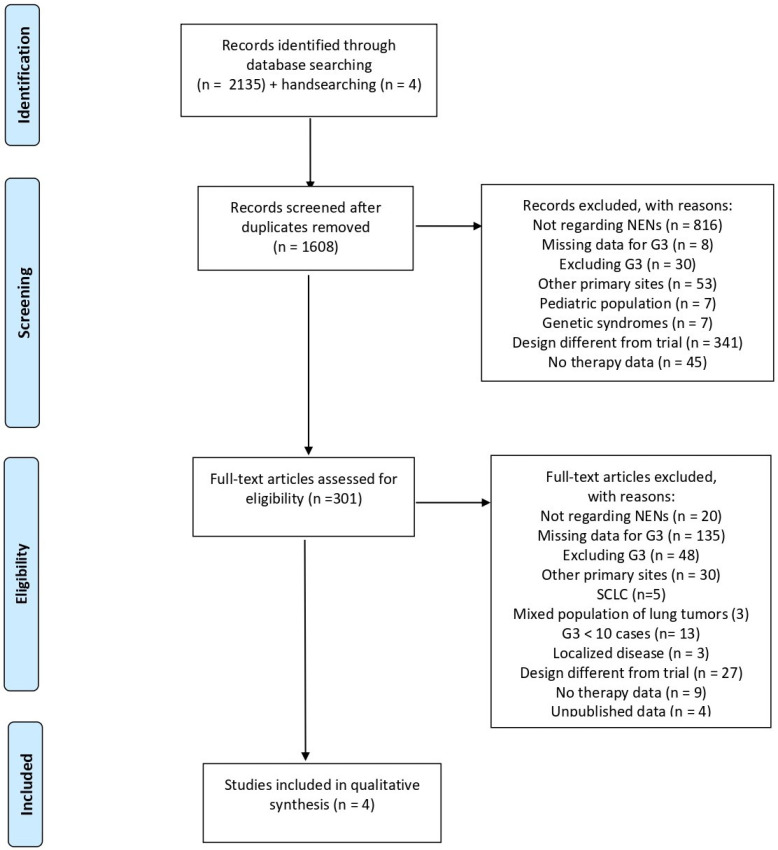
PRISMA flowchart for study selection [22].

Overall, 2139 records were identified through database and hand searches. The study selection process is illustrated in the PRISMA flow diagram presented in Figure 1 [22].

After the removal of 531 duplicate records, a total of 1608 references were screened at the title and abstract level. Following this initial screening phase, 1307 records were excluded because they did not meet the predefined inclusion criteria. Consequently, 301 full-text articles were retrieved and assessed in detail for eligibility. After full-text evaluation, the vast majority of studies were excluded due to reasons such as retrospective design, absence of LCNEC-specific data, non-interventional study design, or lack of relevant clinical outcomes. Ultimately, 4 prospective, non-randomized studies evaluating therapeutic strategies in patients with lung LCNEC were considered eligible and included in the final analysis [23,24,25,26].

**Figure 2:**
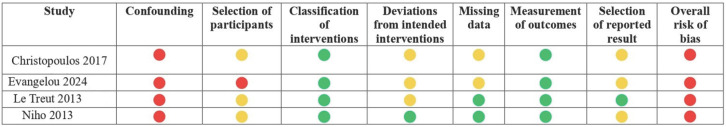
Risk of bias assessment for non-randomized controlled trials (ROBINS-I). Red indicates “high risk of bias”, while yellow and green represents “moderate” and “low risk of bias, respectively [23,24,25,26].

Overall, the risk-of-bias assessment revealed relevant methodological weaknesses across most of the included studies (Figure 2). Indeed, the currently available evidence derives almost exclusively from non-randomized phase II investigations and is therefore inherently more susceptible to bias compared with randomized controlled trials. In particular, the absence of randomization and parallel control arms represents the main methodological limitation, resulting in a substantial risk of confounding and selection bias. In such settings, differences in baseline characteristics, disease burden, or clinical management may influence treatment outcomes and therefore limit the reliability of causal inferences regarding therapeutic efficacy. These limitations are further compounded by the relatively small sample sizes of the included studies, which reduce statistical power and increase the risk of random variation in the observed results. Moreover, the exploratory nature of several trials and the heterogeneity in eligibility criteria, patient populations, and treatment regimens introduce additional variability that complicates the interpretation and comparability of reported outcomes across studies. Differences in diagnostic confirmation, disease stage distribution, and prior treatments may also have contributed to the variability observed in response rates and survival endpoints.

Although outcome measurement and reporting were generally adequate across most studies, and the primary efficacy endpoints were clearly defined, these strengths cannot fully compensate for the structural limitations inherent in the study designs. Consequently, the overall certainty of the available evidence remains limited. For this reason, the reported efficacy and survival outcomes in patients with lung LCNEC should be interpreted with appropriate caution, particularly when attempting to extrapolate these findings to broader clinical practice or to inform treatment guidelines.

Given the limited strength of the available evidence and the substantial methodological variability across the included studies, a formal quantitative meta-analysis could not be reliably performed. In particular, the heterogeneity in study design, patient populations, treatment strategies, and reported outcome measures prevented meaningful statistical pooling of the data.

Therefore, the findings were integrated using a qualitative, narrative synthesis approach, focusing on identifying general patterns and trends in treatment activity across the different therapeutic regimens. Particular attention was given to the reported efficacy outcomes, survival results, and safety profiles, in order to provide a comprehensive overview of the current prospective evidence. This approach allowed us to summarize the available data while acknowledging the limitations inherent to the existing literature.

Across the included studies, ORRs ranged from 34.0% to 50.0% according to the treatment backbone used. Among cytotoxic regimens, cisplatin–irinotecan achieved the highest reported ORR in the centrally confirmed LCNEC subgroup, whereas cisplatin–etoposide yielded a lower but still clinically meaningful activity signal. The combination of carboplatin, paclitaxel, and everolimus was associated with a comparable response rate, but without clear evidence of more durable survival benefit. Median PFS was consistently heterogeneous across studies, ranging from 4.4 months to not reached, while median OS ranged from 8.0 to 12.6 months, overall supporting a pattern of modest and relatively short-lived clinical benefit across treatment strategies. The main results of this descriptive synthesis are presented in Table 1.

### 3.2. Study Results

Three of the included trials investigated first-line chemotherapy regimens, and they were all non-randomized [23,24,25]. The GFPC 0302 trial conducted by Le Treut et al. [23] investigated first-line cisplatin–etoposide chemotherapy in patients with advanced LCNEC. Among the 42 enrolled patients, central pathological review confirmed LCNEC in 29 cases. The median PFS and OS were 5.0 and 8.0 months, respectively. DCR was achieved in a substantial proportion of patients, although ORR was limited. Treatment was associated with considerable toxicity, with nearly 60% of patients experiencing grade 3–4 adverse events, predominantly hematologic, but the data concern the overall study population.

In the multicenter study by Niho et al. [24], cisplatin combined with irinotecan was evaluated in patients initially diagnosed with advanced LCNEC. Following central pathological review, 30 patients were confirmed as having LCNEC and constituted the population of interest. In this subgroup, ORR was 46.7%, with a median PFS of 5.8 months and a median OS of 12.6 months. Although treatment activity was observed, survival outcomes in LCNEC were inferior to those reported in patients reclassified as SCLC in the same study. Hematologic toxicity was frequent, with grade 3–4 neutropenia reported in more than half of the patients, while no treatment-related deaths occurred. Data for the LCNEC is not detailed.

In the multicenter phase II trial by Christopoulos et al. [25], everolimus was combined with carboplatin and paclitaxel as first-line treatment in patients with metastatic lung LCNEC. All enrolled patients had centrally reviewed stage IV disease. The ORR was 45.0%, and DCR was achieved in 74.0% of cases. Median PFS was 4.4 months, while median OS reached 9.9 months. Grade 3–4 toxicities occurred in approximately half of the patients and mainly consisted of cytopenia, infections, and general deterioration in physical condition. Typical everolimus-related adverse events were infrequent and of low grade.

The only study evaluating immunotherapy in lung LCNEC is the LANCE study [26]. This prospective, non-randomized, interventional study by Evangelou et al. evaluated the clinical benefit of adding atezolizumab to first-line platinum-based chemotherapy in 17 patients with metastatic LCNEC. Ten patients received chemo-immunotherapy followed by atezolizumab maintenance, while seven patients received chemotherapy alone. After a median follow-up of 23.3 months, the atezolizumab group showed a significant survival advantage, with a 12-month OS rate of 57.1% versus 14.3% in the control group (*p* = 0.04). The 12-month PFS rate was also higher in the experimental arm (46.7% vs. 14.3%, *p* = 0.05), but these results, although promising, must be read with caution, considering the exploratory design of the study and the very small cohort.

## 4. Discussion

This systematic review highlights a critical disconnect between disease classification and evidence generation: although LCNEC is formally recognized as a distinct clinicopathological entity, it is still managed and investigated largely through extrapolation from SCLC rather than through disease-specific prospective trials. The identified studies are few [23,24,25,26], and predominantly phase II in design, with small sample sizes and heterogeneous inclusion criteria. Importantly, no completed randomized controlled trials evaluating systemic therapies exclusively in advanced pulmonary LCNEC were identified. As a consequence, LCNEC remains a biologically defined entity without a correspondingly dedicated therapeutic evidence base, reinforcing its status as a ‘classified but not clinically individualized’ malignancy.

Across the prospective trials included in this review, platinum-based chemotherapy represents the backbone of first-line systemic treatment for advanced LCNEC, reflecting long-standing extrapolation from SCLC treatment paradigms [4]. Platinum-etoposide and platinum-irinotecan regimens have demonstrated ORR rates ranging from approximately 38% to 55%, indicating that LCNEC retains a degree of chemosensitivity [23,24]. However, clinical benefit appears limited in duration, with median PFS consistently below six months and median OS rarely exceeding one year, highlighting the inability of cytotoxic chemotherapy alone to substantially modify the natural history of advanced disease [3].

Treatment-related toxicity across prospective studies was substantial and broadly comparable to that observed with intensive chemotherapy regimens used in SCLC, particularly with respect to grade 3–4 hematological adverse events [23,24,25]. Given the modest survival outcomes observed across prospective studies, these toxicity profiles raise important concerns regarding the overall risk–benefit balance of aggressive cytotoxic strategies in a patient population frequently diagnosed with advanced-stage disease and often characterized by limited physiological reserve. In this context, treatment-related adverse events—particularly hematological toxicities and treatment interruptions—may significantly affect patients’ tolerance to therapy and potentially compromise the feasibility of subsequent treatment lines. These considerations are particularly relevant in metastatic LCNEC, where therapeutic goals often focus not only on prolonging survival but also on preserving quality of life and maintaining functional status. To date, no prospective study has demonstrated a clear survival advantage associated with the intensification or modification of platinum-based chemotherapy backbones in LCNEC. Although different platinum-based combinations have been explored, the available evidence suggests that improvements in response rates have not translated into meaningful gains in progression-free or overall survival. Consequently, the optimal systemic treatment strategy for advanced LCNEC remains uncertain, and current therapeutic approaches largely continue to rely on regimens historically extrapolated from the management of small-cell lung cancer.

Based on the limited prospective evidence currently available, platinum-based chemotherapy regimens remain the systemic treatment with the most consistent prospective activity signal in advanced LCNEC. Both platinum–etoposide and platinum–irinotecan combinations have demonstrated response rates in the range of approximately 35–50% in prospective studies. However, the biological heterogeneity of LCNEC suggests that a single therapeutic strategy may not be appropriate for all patients. In the absence of prospective molecularly stratified trials, treatment selection in clinical practice often relies on clinicopathological judgement, with SCLC-like regimens frequently preferred for tumors displaying classical neuroendocrine morphology and high proliferative activity [13,27,28].

Overall, the paucity of prospective interventional studies in LCNEC reflects several structural challenges inherent to the study of this rare malignancy. LCNEC accounts for a small fraction of lung cancers, resulting in slow patient accrual and limited feasibility of large, adequately powered randomized trials [3,5]. These challenges are further compounded by diagnostic complexity, evolving pathological criteria, and frequent misclassification with SCLC or other high-grade NENs [1].

Several prospective studies included in this review reported substantial diagnostic reclassification following central pathology review, underscoring not only diagnostic complexity but also the potential fragility of existing clinical datasets, where misclassification may have directly influenced reported efficacy and survival outcomes [23,24]. This observation highlights the importance of seeking a second opinion in clinical practice, particularly when expertise in the field is limited or when dealing with borderline cases that are difficult to define precisely [16,29,30]. Diagnostic heterogeneity introduces additional confounding and limits the generalizability of reported outcomes, particularly in small, single-arm studies. These issues have historically favored feasibility-driven trial designs at the expense of methodological robustness, further weakening the translational value of available evidence.

As a result of these limitations, the current therapeutic landscape of advanced lung LCNEC remains largely empirical. Treatment decisions continue to rely on extrapolation from SCLC rather than on LCNEC-specific data. Emerging molecular studies have demonstrated that LCNEC is a biologically heterogeneous entity, encompassing subsets with genomic features resembling either SCLC or non-small-cell lung cancer, with potential implications for treatment responsiveness [5]. Genomic analyses have identified at least two major molecular subsets: a SCLC-like group characterized by TP53 and RB1 co-inactivation, and a NSCLC-like group enriched for alterations involving STK11, KEAP1, KRAS, and related pathways. This biological heterogeneity may partially explain the inferior and variable outcomes observed when uniform SCLC-based regimens are applied across all LCNEC cases [3,24]. Importantly, none of the prospective interventional studies included in this review incorporated molecular stratification in trial design, thereby applying uniform therapeutic strategies to a diverse disease.

Beyond cytotoxic chemotherapy, prospective evaluation of targeted therapies and immunotherapy-based strategies in pulmonary LCNEC remains extremely limited. The addition of everolimus to platinum–taxane chemotherapy showed acceptable response rates but failed to translate into durable survival benefit, underscoring the challenges of translating biological rationale into clinically meaningful outcomes [25]. More recently, a small, real-world, prospective pilot study has suggested a potential survival advantage with the incorporation of immune checkpoint inhibitors into first-line chemotherapy; however, these findings remain exploratory and are subject to selection bias, limited sample size, and a non-randomized design [26]. Evidence supporting immunotherapy in LCNEC has also emerged indirectly from basket trials enrolling heterogeneous populations of neuroendocrine malignancies [31]. While such studies have contributed to hypothesis generation and demonstrated biological activity across tumor types, the absence of fully disaggregated LCNEC-specific efficacy and survival outcomes substantially limits their applicability to pulmonary LCNEC.

Importantly, the limited number of prospective studies published does not reflect an absence of ongoing research activity. An increasing number of registered interventional trials specifically targeting pulmonary LCNEC indicates growing recognition of the unmet clinical need in this population (e.g., NCT06393816, NCT06418087, NCT05470595). Most ongoing studies are phase II, single-arm trials evaluating the addition of immune checkpoint inhibitors to platinum–etoposide chemotherapy, largely extrapolating from therapeutic advances achieved in extensive-stage SCLC [5]. Nevertheless, the predominance of non-randomized designs and reliance on historical controls raise concerns regarding the interpretability and generalizability of forthcoming results. Without systematic central pathology review and integration of molecular stratification, there is a risk that emerging therapeutic strategies will continue to be applied empirically, perpetuating the methodological limitations that have historically characterized LCNEC research [1,5]. Most ongoing studies are phase II, single-arm trials that largely replicate historical SCLC-based strategies with the addition of immune checkpoint inhibitors, raising concern that future evidence may continue to suffer from the same structural limitations observed in prior LCNEC research. Future clinical trials in LCNEC should incorporate several methodological elements to enhance interpretability and translational value, including mandatory central pathology review, prospective molecular stratification based on biologically relevant subtypes, and clinically meaningful endpoints beyond ORR (e.g., duration of response, patient-reported outcomes, and quality-of-life measures). Furthermore, integrated translational research programs should be aimed at identifying predictive biomarkers of therapeutic response and resistance.

## 5. Conclusions

Collectively, these findings reveal a structural gap between classification and therapeutic evidence generation in pulmonary LCNEC, underscoring the urgent need for dedicated, molecularly informed, disease-specific clinical trials. Closing this gap will require coordinated international collaboration, harmonized diagnostic standards with mandatory central pathology review, prospective molecular stratification, and innovative biomarker-driven trial designs tailored to rare and biologically heterogeneous malignancies. Integration of clinically meaningful endpoints and embedded translational research programs will be essential to move beyond empirical treatment selection and toward a genuinely disease-specific therapeutic strategy for pulmonary LCNEC. Without such efforts, LCNEC will remain a diagnostically refined but therapeutically underdeveloped entity, with clinical decisions continuing to rely on extrapolation rather than on robust, disease-specific evidence.

## Figures and Tables

**Table 1:** Presentation of the studies included in the systematic review.

Study	Study Design	*n* (%)	Therapy	Response	Survival Data (Mos)	Toxicity
Christopoulos [25]	Non-randomized, multicenter phase II trial	49 (100)	- Induction: 5 mg everolimus daily + paclitaxel 175 mg/m^2^ + carboplatin AUC 5 every 3 weeks (max 4 cycles) - Maintenance: everolimus 5 mg daily	- ORR: 45.0% (95% CI 31–60%; CR 0, PR 22) - DCR: 74.0% (95% CI 59–85%)	- Median PFS: 4.4 (95% CI: 3.2–6) - Median OS: 9.9 (95% CI: 6.9–11.7)	- Grade ≥ 3: 51.0% (mainly cytopenias 24%, infections 10%, GI 8%, general deterioration 8%) - Everolimus-related AE: stomatitis, rash, mainly grade 1–2
Evangelou [26]	Non-randomized, comparative, single-center, pilot study	17 (100)	- Induction: Carboplatin + etoposide + atezolizumab (n = 10) vs. carboplatin + etoposide (n = 7) - Maintenance: atezolizumab in responders	ORR: 50.0% (chemo + atezolizumab) vs. 42.9% (chemo alone)	- Median PFS: not reached vs. 5.2 - Median OS: not reached vs. 8.2	- Grade 3: 2 cases (skin toxicity, pneumonitis) - No grade 4, no treatment-related deaths
Le Treut [23]	Non-randomized, multicentre, single-arm, phase II trial	29 (69.0) *	Cisplatin 80 mg/m^2^ on day 1 (D1) + etoposide 100 mg/m^2^ on D1, D2, and D3 (at 21-day intervals, max 6 cycles)	- ORR: 34.0% (CR 0, PR 10) - DCR: 65.0%	- Median PFS: 5.0 (95% CI 4.0–7.9) - Median OS: 8.0 (95% CI 3.7–7.9) †	‡
Niho [24]	Non-randomized, multicentre, single-arm, phase II trial	30 (68.2) *	Irinotecan 60 mg/m^2^ (days 1, 8, 15) + cisplatin 60 mg/m^2^ (day 1) every 4 weeks, up to 4 cycles	- ORR: 46.7% (95% CI 28.3–65.7%; CR 0, PR 14) - DCR: 80.0%	- Median PFS: 5.8 (3.8–7.8) - Median OS: 12.6 (9.3–16.0)	‡

* Percentages represent the proportion of patients with LCNEC confirmed after central pathology review within the initially enrolled study population; † This interval appears numerically inconsistent with the reported median and may reflect a typographical error in the source article; ‡ Toxicity data were reported for the overall study populations, but not separately for the centrally confirmed LCNEC subgroup; ORR: objective response rate (CR + PR); CR: complete response; PR: partial response; DCR: disease control rate (CR + PR + SD); SD: stable disease; PFS: progression-free survival; OS: overall survival; GI: gastrointestinal; AE: adverse events.

## Data Availability

No new data were created or analyzed in this study.

## Supplementary Materials

The following supporting information can be downloaded at: https://www.mdpi.com/article/10.3390/cancers18060964/s1, Table S1: World Health Organization (WHO) Classification 2021 for lung neuroendocrine neoplasms; Supplementary Material S1: Search strategies.

## Abbreviations

The following abbreviations are used in this manuscript:

LCNEC	Large cell neuroendocrine carcinoma
NENs	Neuroendocrine neoplasms
SCLC	Small cell lung carcinoma
WHO	World Health Organization
ORR	Objective response rate
DCR	Disease control rate
PFS	Progression-free survival
OS	Overall survival
