# Supplementary material for: Interventional Clinical Trials in Metastatic Pulmonary Large-Cell Neuroendocrine Carcinoma: A Systematic Review of Prospective, Interventional Trials
Source: Cancers (Basel). 2026 Mar 17;18(6):964. doi: 10.3390/cancers18060964 (PMC13024530; doi:10.3390/cancers18060964)
Supplement: Supplementary file 1 [file cancers-18-00964-s001.zip › Supplementary Material S1.pdf]

Supplementary Material S1: Search strategies

PUBMED

(("Neuroendocrine Tumors"[Mesh] OR "Carcinoma, Neuroendocrine"[Mesh] OR (neuroendocrine[tiab] AND (tumor*[tiab] OR tumour*[tiab] OR carcinoma*[tiab] OR neoplasm*[tiab])) OR NET[tiab] OR NEN[tiab] OR NEC[tiab]) AND ("grade 3"[tiab] OR G3[tiab] OR "high grade"[tiab] OR "high-grade"[tiab])) AND ("Randomized Controlled Trial"[Publication Type] OR "Clinical Trial"[Publication Type] OR randomized[tiab] OR randomised[tiab] OR trial[tiab]) NOT (animals[mh] NOT humans[mh])

09/08/2025 1163 papers

SCOPUS

TITLE-ABS-KEY((neuroendocrine AND (tumor* OR tumour* OR carcinoma* OR neoplasm*) OR NET OR NEN OR NEC) AND ("grade 3" OR G3 OR "high grade" OR "high-grade") AND ("clinical trial" OR "randomized controlled trial" OR "controlled clinical trial" OR randomised OR randomized OR trial)) AND NOT TITLE-ABS-KEY(animal*)

09/08/2025 733 papers

WEB OF SCIENCE

#1 TS=(neuroendocrine OR "NET" OR "NEN" OR "NEC")

#2 TS=(tumor OR tumour* OR carcinoma* OR neoplasm*)*

#3 TS=("grade 3" OR G3 OR "grade III" OR "high grade" OR "high-grade")

#4 TS=("clinical trial" OR randomized OR randomised OR "controlled clinical trial")

09/08/2025 239 papers
