# Supplementary material for: Interventional Clinical Trials in Metastatic Pulmonary Large-Cell Neuroendocrine Carcinoma: A Systematic Review of Prospective, Interventional Trials
Source: Cancers (Basel). 2026 Mar 17;18(6):964. doi: 10.3390/cancers18060964 (PMC13024530; doi:10.3390/cancers18060964)
Supplement: Supplementary file 1 [file cancers-18-00964-s001.zip › Supplementary Table S1.pdf]

cancers

Supplementary Table S1. World Health Organization (WHO) Classification 2021 for lung neuroendocrine neoplasms.

Neuroendocrine neoplasm	Diagnostic criteria
Neuroendocrine tumor/Carcinoid tumor	
Typical carcinoid	< 2 mitoses/2 mm2 and no necrosis
Atypical carcinoid*	2–10 mitoses/2 mm2 and/or necrosis (usually punctate)
Neuroendocrine carcinoma	
Small cell carcinoma (SCLC)	>10 mitoses/2 mm2 , often necrosis and small cell cytomorphology
Large cell neuroendocrine carcinoma (LCNEC)	>10 mitoses/2 mm2 , virtually always necrosis and large cell cytomorphology

* A novel category of with elevated mitotic counts (atypical carcinoid morphology + a mitotic count >10 mitoses per 2 mm2 and/or Ki-67 > 30%) has been proposed

 https://doi.org/10.3390/xxxxx
